# A Case Cured with Argentum Nitricum

**Published:** 1893-02

**Authors:** W. P. Wesselhœft

**Affiliations:** Boston, Mass.


					﻿A CASE CURED WITH ARGENTUM NITRICUM.
W. P. Wesselhceft, M. D., Boston, Mass.
G. H. B., set. forty-three, dark blonde, has always enjoyed
-good health till May 30th, 1890.
On this day a small boil appeared on lower portion of left
oalf. This very rapidly increasing in size, assuming a dark,
almost black color. In about three weeks a large slough was
thrown off, the size of the palm of a hand. Nearly at the same
time a large vesicle or bulla appeared on the left cheek, which
went through a similar process as the one on the calf. Both
places have red cicatrices with some loss of tissue. During this
time he was treated by one of our most prominent dermatologists,
mainly with external applications of Zinc.
The process of healing of both sores occupied about two
months.
Immediately after the healing of these ulcers a prickling
sensation commenced in the ball of right foot and in the finger
ends of both hands. For this a mixture of Strychnia and
Phosph.-acid, from a celebrity in Philadelphia, was prescribed.
Nevertheless, the prickling and numbness rapidly increased;
his gait became unwieldy and clumsy, with increasing difficulty
■of raising his feet.
On September 4th, 1890, he applied to me, when the follow-
ing symptoms were recorded :
Numbness of both hands, especially of fingers, so that he finds
it impossible to button his clothing. The grip of hands is weak,
and rapidly growing more so. Finds great difficulty in writing
on account of clumsiness of hand. Drops things frequently
which he has grasped.
Numbness of feet and legs extending to thighs. Left leg
worse. This numbness has gradually crept upward for two
months. Difficulty of raising feet from ground. Has no patel-
lar reflex.
After walking one-eighth of a mile legs give out from under
him. He drags his heels while walking. Difficulty of balanc-
ing himself. Walks with feet wide apart. Much worse with
eyes closed, and is helpless in the dark. Toes of right foot
drawn spasmodically downward, drawing contraction in both
calves.
Sleep, appetite, stools are normal.
Has old protruding (external) piles for twenty years, which
give him no inconvenience, except when irritated by walking in
warm weather.
Is a bachelor, who has led a pure and temperate life, and ac-
customed to athletic exercises.
On the 4th of September, 1890, gave him two doses of Argent-
nitr.cm, one to be taken in the morning and the other at
night.
On the 18th of September reported increased contraction in
calves and much more numbness during the first week. During
the last week his feet better; less numbness of fingers, and calves
less contracted.
On this day I met his former physician, the dermatologist,
and told him the main features of the case. He said that he could
now account for the peculiar gangrenous ulcers, these being due
to a serious lesion of the spine.
September 25th.—Reports marked improvement in sense of
touch. He writes more easily and his gait has improved. He
now complains of a sense of weakness and pain in lower lumbar
region.
Improvement continued till October 30th. Reports more
weakness and pain in back. The numb sensation in limbs
has steadily improved, but for the last week has noticed no
change. He now received a dose of Argent-nitr.dm.
Steady and great improvement followed till
December 5th.—Reports more pain and weakness in lower
lumbar region. Numbness of upper and lower extremities has
disappeared, with the exception of a slight feeling in the tip of
the right toe. Argent-nitr.cmm, one dose.
Continued improvement followed, and he was discharged well
on the 11th of May, 1891. He has remained so, with constant
attention to arduous duties devolving upon a president of one
of our largest insurance companies, and I have had no occasion
to prescribe for him since.
What would have become, of this poor fellow under the treat-
ment of his previous medical advisers?
CLINICAL CASES.
Jennie Medley, M. D., Philadelphia, Pa.
Case I.—Mrs. L., aet. sixty-two. This patient for two years
past had been suffering with uterine hemorrhages. When she
did not have a discharge of blood, had a constant discharge of
greenish or yellowish offensive water, with an occasional cheesy
lump. She had borne nine children, had ceased menstruating
about her fifty-fifth year—but, within last two years flow has
returned. For the last six months her health is failing; is
naturally very active, but finds her strength rapidly giving way.
Symptoms.—Great heaviness in the lower extremities; tired
all over ; it is a great effort to go up-stairs; very low spirited ;
grieved because she could not do her housework; bowels con-
stipated—mus’t strain until weak before the bowels moved.
Appetite poor; there were the burning, sticking pains through
the cervix; on examination found left side of the cervix par-
tially destroyed. Tried the speculum, but at all times the dis-
charge of blood was so great that I could see nothing. Digital
examination, however, revealed the condition. On the above
symptoms she received a dose of Sil.72m. In about a month she
was in wonderfully better spirits, was very much stronger, and
the offensive odor of the discharge had entirely disappeared.
From a repetition of the 72m to the millionth within a year, of
which she received two doses each, she suffered very little pain,
the case apparently being at a standstill. Her principal suffer-
ing during this time was great mental depression. Until the
end of six months after she received the last dose of Sil.m her
symptoms remained the same; at the close of six months she
begun to have dyspnoea; couldn’t sleep in a room where the
least heat had been admitted—must have windows open in addi-
tion to being in a cold room. She was still kept on S-l.; this
state of affairs was permitted to go on for some days. Finally,
was sent for in a great hurry; arrived at the house and learned
that first one and then another member of the family had been
taking their turn at fanning her all night. When they ceased
to fan she gasped for breath; this with the fan-like motion of
the alee nasi led to a prescription of Carbo-veg.cm which in
fifteen minutes relieved her; after six doses of Carbo-veg. at
such intervals as were made necessary by the return of uncom-
fortable symptoms, the CM proved no longer palliative, a
dose of the millionth of the same remedy was given. This re-
lieved promptly. At the close of three weeks the symptoms
had changed, the burning returned violently. Carbo-veg.m
was again given without effect. In addition to the burning she
had thirst, little and often ; great restlessness ; was thirsty, but
a small quantity satisfied, and was followed by great distress in
the stomach. Ars.90m was then given. I felt so sure that
it would help that I left, thinking in a short time all would be
well,. The Ars. was administered at 1 p. m. Sunday, the
next morning, at nine o’clock, I was sent for. She had passed
the night in agony, and begged for Morphine. I went home,
looked over the case, and sent her again a dose of the 72m of
Silicea. It acted like magic. She fell asleep in one hour, and
from that time on she had to.be shaken up, to arouse her suffi-
ciently to take nourishment. She had no pain and only com-
plained of being so tired and sleepy. For twenty-four hours
before she died she lay dead asleep, and died without pain.
Her son, who is not a strict homoeopath, could scarcely believe
that one could die with such a disease with so little pain. The
absence of odor also surprised him very much.
Case II.—Mrs. W., aet. thirty-two, mother of four children,
three months advanced in pregnancy. From the beginning of
her pregnant condition had suffered with constipation, would be
worn out after a stool, she was obliged to go to bed; during the
three months of pregnancy didn’t have what she would call a
good movement. At last she took la grippe which Rhus-t., the
lm, helped in twenty-four hours. She felt so well that she was
going to get up, when suddenly she was taken with a hemor-
rhage, not violent, but very steady; the blood was bright red
with dark clots. She had no pain, but terrific nausea. She had
been in this condition two hours when I arrived. I immedi-
ately gave her a dose of Ipecac., but it neither brought on pains
nor stopped the hemorrhage, so all to be done was to wait. The
hemorrhage continued and still symptoms did not change. After
several hours of watching and waiting she became weak, a pro-
fuse cold sweat covered her body, wanted to make her will im-
mediately, would not be persuaded not to. Aeon.2® was given
in a broken dose, three teaspoonfuls of water every fifteen min-
utes ; pains come on and the foetus and all, which was of five
weeks’ development at most, was expelled. She was now ex-
ceedingly weak and immediately after delivery I gave a dose of
Sul.®”*. After several hours she became violently thirsty. She
dropped to sleep and in a few minutes woke up with violent
pain in the heart. This made me think of Lach., but felt I
would be sure of it. I then waited until she fell asleep again.
She awoke in the same manner. I then gave her Lach. This
relieved her. After taking the Lach, she again slept, but it was
only for a very short time, and the pain was still quite severe but
less sharp. Two of my brother physicians suggested remedies :
one suggested Sepia and the other Pyrogen. Having given Sepia
myself in the beginning for the constipation, I knew it would be
of no avail now, and I felt certain that Pyrogen would be the
remedy to pull her through if it were possible. Accordingly,
after watching the temperature gradually rise to 101|°, eight
hours after the Lach. I gave Pyrogen50”1; that was about 2 p. m.
At 5 p. m. she urinated naturally. One hour later she had a large
stool, the first hard lump was removed with the fingers, she was
too weak to strain it away. Two hours later she had another
soft stool without the least trouble. When the examination
was first made at the beginning of the abortion the rectum was
piled full of hard stool. These two symptoms encouraged me
very much; the Pyrogen wore out every seven hours, which was
also the experience of the physician who suggested it. After
several doses of the 50m had been given the cm was given the
patient. In four days the patient was completely out of danger
and in three weeks was sitting up. In exactly four weeks after
the abortion menstruation came on with a hemorrhage at twelve
o’clock at night. She lost about a quart and a half of blood ;
this was also full of dark clots. The same physician who sug-
gested the Pyrogen now gave Ipecac. I called several hours
after, but the hemorrhage still continued. I gave Pyrogen,
which stopped the hemorrhage and the patient made a good re-
covery. After the first six hours during the abortion I cen-
sured myself for not using mechanical measures, but now I am
satisfied, as I feel quite sure I would have had a post-partum
hemorrhage that I could not manage, being ignorant of how
closely Pyrogen resembled Ipecac, in that respect. I also feel
satisfied that the patient would have died had she not received
Pyrogen, as the only case of septicemia I have ever lost was
one which presented just such symptoms.
The following committee was appointed upon the subject of
Vaccination to report at the next meeting:
Drs. Close, Custis, and A. G. Allan.
MELANCHOLIA.
C. C. Howard, M. D., New York, N. Y.
Case I.—Mrs. B----, set. thirty-nine; dark hair, dark eyes,
swarthy complexion; quick tempered, overbearing, proud;
always enjoyed good health. Was suddenly seized with an
irresistible desire, while sitting in the dentist’s chair, to strike
the dentist in the genital organs. This inclination became so
strong that she excused herself and went home. It caused her
much anxiety. She was afraid of some mental disturbance.
She cried a good deal, became very restless. In about two
weeks she went back to the dentist, having had no like impulse
in the meantime. Was scarcely seated when the same irresist-
ible impulse returned. It grew upon her so rapidly in the next
ten days that every male she met, even her husband, her own
sons, affected her the same way.
Her suffering became unbearable. About six o’clock every
evening there would be a violent aggravation, lasting until bed-
time, when she would generally fall asleep. During the aggra-
vation she would walk the floor, wring her hands, and cry
in the most pitiable manner. Or she would sit on the side of
her bed, and sway herself backward and forward. Her coun-
tenance expressed the most abject suffering. She was sure she
was not curable; she was becoming deranged, and wanted her
children sent away so they would not see her.
She menstruated very profusely every three weeks; large,
dark clots, with soreness of the vulva.
She did not apply to me for a month after the first manifesta-
tion.
Her haughty manner, swarthy complexion, the great mental
anxiety, the wringing of the hands, and the swaying of the body,
.the sexual thoughts, together with the menstrual irregularities,
led me to the selection of Platina, of which I gave her one dose
of the 50mm.
She gradually improved for the next six weeks, menstruating
at the twenty-eight-day period, everything being perfectly nor-
mal, when there was a sudden and violent return of the mental
condition.
The relapse was accompanied by a quite marked disturbance
of the stomach. As soon as the stomach was empty, she suf-
fered from a gnawing hunger and griping pain, relieved by eat-
ing, but the eating caused an oppression.
While talking to her she received some flowers sent by a
friend, and exclaimed, “How very kind, but take them out of
the room, the odor is unbearable!” This symptom, with the
condition of the stomach, relief from eating, and the mental
restlessness, made me think of Graphites, of which I gave her
one dose of the CM potency.
Now there was no change in her condition for twenty-one
days, and, then, after a severe aggravation on the twenty-first
night, she awakened completely relieved of all her symptoms.
In three months she became pregnant, and was well during the
period of gestation, and gave birth to a healthy child.
About two months after labor a fear gradually came over her
that she might do harm to her baby I This gave her so much
anxiety that she would not be left alone with the infant. She
was restless, melancholy, and tearful. There was a return of
the stomach symptoms, and the same aggravation from the odor
of flowers. She received another dose of Graphites, this time
■dissolved in twelve teaspoonfuls of water. She improved im-
mediately, and has had no return up the present time—more
than six months.
Case II.—Mrs. S-------, set. thirty-seven, mild, gentle disposi-
tion, easily worried, light complexion. Great tendency to grow
fat; perspires on the slightest exertion and at night during her
sleep. Last August had a very difficult labor, in which she
sustained a severe laceration of the cervix, and a slight one of the
perineum, which left her in a melancholy condition.
She was doubtful as to a future state; feared she was losing
her reason, and that others would observe her confusion of
mind. Loss of memory; dreaded seeing people—and left the
room, if possible, when any one called. Very melancholy, de-
pressed, and tearful. Continually worried over imaginary evils
that might befall her family.
There was great difficulty in walking; the legs were stiff,
gait tottering, knees would give out in walking, and she.would
fall unless support was near. There was dull, heavy, aching
pain at the base of brain extending down the spine. Calc-
•carb.cm, one dose. She began to improve in about ten days, and
there was a complete restoration to health in two months.
Tabes Dorsalis.
Case III.—Mr. A., set. sixty-two, hard-working business-
man of temperate habits, but never took any exercise. Had been
sick for two years before consulting me, having had all sorts of
allopathic abominations, even to the hot iron.
Presented the following conditions :—Inability to walk with-
out looking at his feet—would stagger and fall in the dark, or
on lifting his eyes from the ground. Walked with his feet far
apart. Sensation as if walking on balls. Paroxysms of pain in
thighs, legs, and feet; heavy, dull ache at times, and again sharp
and lancinating. The paroxysms would last two or three days,
and come about every week. They were worse in damp-
weather. There was a sensation in the feet as if being squeezed
in a vise. This was particularly felt in the big toe of the left
foot.
His appetite and digestion were good, although he had to be
very cautious—eating to satiety would aggravate the pains.
Craving for clean white rags. His bowels were very much con-
stipated, and would only move when there was a large accumu-
lation and with much straining. The feces were covered with
mucus and streaked with blood. I gave one dose of Alumina49*”*
Although I watched the case very carefully I could not see
the slightest improvement for over nine weeks.
Then the bowels began to move more regularly and the stools
assumed a more healthy appearance. Week by week the pains
grew less and he walked with greater ease.
He gradually became able to walk in the dark, and after six
months was practically well, and has remained so, up to the
present time, about two years and a half after beginning treat-
ment.
				

